# 2-(3-Chloro­anilino)pyridine

**DOI:** 10.1107/S1600536809019941

**Published:** 2009-05-29

**Authors:** Zainal Abidin Fairuz, Zaharah Aiyub, Zanariah Abdullah, Seik Weng Ng

**Affiliations:** a Department of Chemistry, University of Malaya, 50603 Kuala Lumpur, Malaysia

## Abstract

In the title compound, C_11_H_9_ClN, the dihedral angle between the aromatic ring planes is 44.2 (1)° and the bridging C—N—C bond angle is 127.60 (19)°. The amino N—H grouping makes a hydrogen bond to the pyridyl N atom of an adjacent mol­ecule across a center of inversion, generating a hydrogen-bonded dimer.

## Related literature

For the crystal structure of the 4-chloro derivative, see: Fairuz *et al.* (2008 ▶).
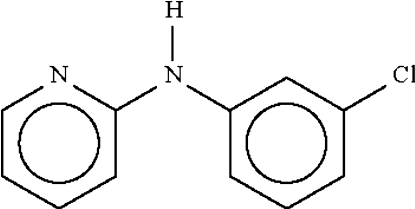


## Experimental

### 

#### Crystal data

C_11_H_9_ClN_2_
*M* *_r_* = 204.65
Triclinic, 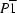

*a* = 3.8954 (1) Å
*b* = 10.7804 (4) Å
*c* = 12.4548 (4) Å
α = 64.932 (2)°
β = 88.004 (2)°
γ = 88.240 (2)°
*V* = 473.40 (3) Å^3^
*Z* = 2
Mo *K*α radiation
μ = 0.36 mm^−1^
*T* = 119 K
0.40 × 0.05 × 0.02 mm

#### Data collection

Bruker SMART APEX diffractometer
Absorption correction: multi-scan (*SADABS*; Sheldrick, 1996 ▶) *T*_min_ = 0.870, *T*_max_ = 0.993
5923 measured reflections
2064 independent reflections
1807 reflections with *I* > 2σ(*I*)
*R*_int_ = 0.019

#### Refinement

*R*[*F* ^2^ > 2σ(*F* ^2^)] = 0.047
*wR*(*F* ^2^) = 0.133
*S* = 1.07
2064 reflections
131 parameters
1 restraint
H atoms treated by a mixture of independent and constrained refinement
Δρ_max_ = 0.37 e Å^−3^
Δρ_min_ = −0.29 e Å^−3^

### 

Data collection: *APEX2* (Bruker, 2008 ▶); cell refinement: *SAINT* (Bruker, 2008 ▶); data reduction: *SAINT*; program(s) used to solve structure: *SHELXS97* (Sheldrick, 2008 ▶); program(s) used to refine structure: *SHELXL97* (Sheldrick, 2008 ▶); molecular graphics: *X-SEED* (Barbour, 2001 ▶); software used to prepare material for publication: *publCIF* (Westrip, 2009 ▶).

## Supplementary Material

Crystal structure: contains datablocks global, I. DOI: 10.1107/S1600536809019941/tk2464sup1.cif

Structure factors: contains datablocks I. DOI: 10.1107/S1600536809019941/tk2464Isup2.hkl

Additional supplementary materials:  crystallographic information; 3D view; checkCIF report

## Figures and Tables

**Table 1:** Hydrogen-bond geometry (Å, °)

*D*—H⋯*A*	*D*—H	H⋯*A*	*D*⋯*A*	*D*—H⋯*A*
N1—H1⋯N2^i^	0.88 (1)	2.18 (1)	3.042 (3)	167 (3)

Symmetry code: (i) 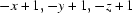.

**Fig. 1.**
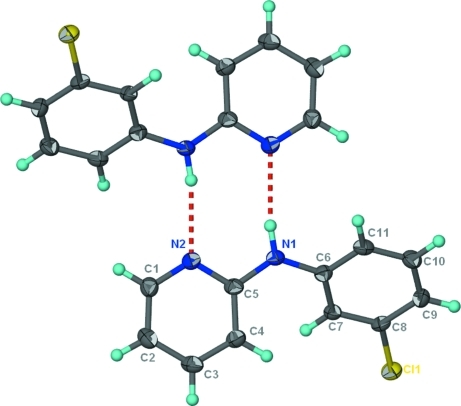
Thermal ellipsoid plot (Barbour, 2001) of the hydrogen-bonded (dashed lines) centrosymmetric dimer {C11H9ClN2}2 with molecules drawn at the 70% probability level; hydrogen atoms are drawn as spheres of arbitrary radius.

## supplementary crystallographic information

### Experimental

2-Chloropyridine (0.5 ml, 5.28 mmol) and 3-chloroaniline (0.67 g, 5.28 mmol) were heated at 423–433 K for 3 h. The solid was dissolved in water and extracted with ether. The ether extract was dried over sodium sulfate. The solvent was evaporated and the product recrystallized from ethanol to yield pale-purple crystals.

### Refinement

Carbon-bound H-atoms were placed in calculated positions (C—H 0.95 Å) and were included in the refinement in the riding model approximation with *U*(H) fixed at 1.2*U*(C). The amino H-atom was located in a difference Fourier map and was refined with a distance restraint of N–H 0.88±0.01 Å; the isotropic temperature factor were refined.

### Crystal data

**Table:**

C_11_H_9_ClN_2_	*Z* = 2
*M**_r_* = 204.65	*F*(000) = 212
Triclinic, *P*1	*D*_x_ = 1.436 Mg m^−^^3^
Hall symbol: -P 1	Mo *K*α radiation, λ = 0.71073 Å
*a* = 3.8954 (1) Å	Cell parameters from 1691 reflections
*b* = 10.7804 (4) Å	θ = 3.2–27.7°
*c* = 12.4548 (4) Å	µ = 0.36 mm^−^^1^
α = 64.932 (2)°	*T* = 119 K
β = 88.004 (2)°	Prism, pale purple
γ = 88.240 (2)°	0.40 × 0.05 × 0.02 mm
*V* = 473.40 (3) Å^3^	

### Data collection

**Table:**

Bruker SMART APEX diffractometer	2064 independent reflections
Radiation source: fine-focus sealed tube	1807 reflections with *I* > 2σ(*I*)
graphite	*R*_int_ = 0.019
ω scans	θ_max_ = 27.5°, θ_min_ = 1.8°
Absorption correction: multi-scan (*SADABS*; Sheldrick, 1996)	*h* = −5→5
*T*_min_ = 0.870, *T*_max_ = 0.993	*k* = −13→14
5923 measured reflections	*l* = −16→16

### Refinement

**Table:**

Refinement on *F*^2^	Primary atom site location: structure-invariant direct methods
Least-squares matrix: full	Secondary atom site location: difference Fourier map
*R*[*F*^2^ > 2σ(*F*^2^)] = 0.047	Hydrogen site location: inferred from neighbouring sites
*wR*(*F*^2^) = 0.133	H atoms treated by a mixture of independent and constrained refinement
*S* = 1.07	*w* = 1/[σ^2^(*F*_o_^2^) + (0.0578*P*)^2^ + 0.8*P*] where *P* = (*F*_o_^2^ + 2*F*_c_^2^)/3
2064 reflections	(Δ/σ)_max_ = 0.001
131 parameters	Δρ_max_ = 0.37 e Å^−^^3^
1 restraint	Δρ_min_ = −0.29 e Å^−^^3^

### Special details

**Table:**

Geometry. All e.s.d.'s (except the e.s.d. in the dihedral angle between two l.s. planes) are estimated using the full covariance matrix. The cell e.s.d.'s are taken into account individually in the estimation of e.s.d.'s in distances, angles and torsion angles; correlations between e.s.d.'s in cell parameters are only used when they are defined by crystal symmetry. An approximate (isotropic) treatment of cell e.s.d.'s is used for estimating e.s.d.'s involving l.s. planes.

### Fractional atomic coordinates and isotropic or equivalent isotropic displacement parameters (Å^2^)

**Table:**

	*x*	*y*	*z*	*U*_iso_*/*U*_eq_	
Cl1	0.67761 (16)	−0.15289 (6)	0.94724 (5)	0.0242 (2)	
N1	0.7040 (6)	0.3450 (2)	0.62528 (17)	0.0224 (5)	
H1	0.654 (8)	0.390 (3)	0.5500 (11)	0.024 (7)*	
N2	0.5081 (5)	0.5439 (2)	0.63195 (17)	0.0207 (4)	
C1	0.4688 (6)	0.6248 (2)	0.6895 (2)	0.0215 (5)	
H1A	0.3597	0.7118	0.6492	0.026*	
C2	0.5768 (7)	0.5897 (3)	0.8032 (2)	0.0237 (5)	
H2	0.5413	0.6501	0.8404	0.028*	
C3	0.7401 (7)	0.4624 (2)	0.8620 (2)	0.0230 (5)	
H3	0.8192	0.4351	0.9402	0.028*	
C4	0.7861 (6)	0.3767 (2)	0.8060 (2)	0.0206 (5)	
H4	0.8982	0.2901	0.8443	0.025*	
C5	0.6623 (6)	0.4211 (2)	0.69013 (19)	0.0186 (5)	
C6	0.8088 (6)	0.2076 (2)	0.6660 (2)	0.0185 (5)	
C7	0.7102 (6)	0.1066 (2)	0.77788 (19)	0.0179 (5)	
H7	0.5747	0.1306	0.8314	0.022*	
C8	0.8122 (6)	−0.0275 (2)	0.80912 (19)	0.0179 (5)	
C9	1.0038 (6)	−0.0685 (2)	0.7336 (2)	0.0209 (5)	
H9	1.0677	−0.1619	0.7565	0.025*	
C10	1.0995 (6)	0.0324 (3)	0.6223 (2)	0.0225 (5)	
H10	1.2316	0.0075	0.5687	0.027*	
C11	1.0045 (6)	0.1685 (2)	0.5891 (2)	0.0201 (5)	
H11	1.0731	0.2357	0.5132	0.024*	

### Atomic displacement parameters (Å^2^)

**Table:**

	*U*^11^	*U*^22^	*U*^33^	*U*^12^	*U*^13^	*U*^23^
Cl1	0.0325 (4)	0.0186 (3)	0.0169 (3)	−0.0002 (2)	−0.0003 (2)	−0.0032 (2)
N1	0.0351 (12)	0.0172 (10)	0.0128 (9)	0.0037 (8)	−0.0029 (8)	−0.0044 (8)
N2	0.0263 (11)	0.0173 (9)	0.0156 (9)	0.0010 (8)	0.0006 (8)	−0.0043 (7)
C1	0.0234 (12)	0.0194 (11)	0.0205 (11)	0.0009 (9)	0.0027 (9)	−0.0076 (9)
C2	0.0287 (13)	0.0228 (12)	0.0222 (12)	−0.0034 (10)	0.0036 (10)	−0.0119 (10)
C3	0.0297 (13)	0.0224 (12)	0.0158 (11)	−0.0051 (10)	−0.0008 (9)	−0.0069 (9)
C4	0.0225 (12)	0.0183 (11)	0.0194 (11)	−0.0007 (9)	−0.0038 (9)	−0.0061 (9)
C5	0.0227 (12)	0.0169 (11)	0.0135 (10)	−0.0034 (9)	0.0011 (8)	−0.0038 (8)
C6	0.0192 (11)	0.0185 (11)	0.0173 (11)	0.0007 (9)	−0.0028 (8)	−0.0071 (9)
C7	0.0191 (11)	0.0204 (11)	0.0139 (10)	0.0021 (9)	−0.0005 (8)	−0.0070 (9)
C8	0.0198 (11)	0.0166 (10)	0.0139 (10)	−0.0013 (8)	−0.0022 (8)	−0.0029 (8)
C9	0.0227 (12)	0.0180 (11)	0.0224 (11)	0.0032 (9)	−0.0038 (9)	−0.0091 (9)
C10	0.0209 (12)	0.0286 (13)	0.0201 (11)	0.0026 (9)	−0.0002 (9)	−0.0126 (10)
C11	0.0202 (12)	0.0244 (12)	0.0145 (10)	−0.0011 (9)	−0.0004 (8)	−0.0071 (9)

### Geometric parameters (Å, °)

**Table:**

Cl1—C8	1.752 (2)	C4—C5	1.411 (3)
N1—C5	1.376 (3)	C4—H4	0.9500
N1—C6	1.400 (3)	C6—C11	1.396 (3)
N1—H1	0.880 (10)	C6—C7	1.406 (3)
N2—C5	1.345 (3)	C7—C8	1.378 (3)
N2—C1	1.346 (3)	C7—H7	0.9500
C1—C2	1.380 (3)	C8—C9	1.386 (3)
C1—H1A	0.9500	C9—C10	1.398 (3)
C2—C3	1.397 (3)	C9—H9	0.9500
C2—H2	0.9500	C10—C11	1.386 (3)
C3—C4	1.378 (3)	C10—H10	0.9500
C3—H3	0.9500	C11—H11	0.9500
			
C5—N1—C6	127.60 (19)	N1—C6—C11	118.1 (2)
C5—N1—H1	114.3 (19)	N1—C6—C7	123.0 (2)
C6—N1—H1	118.1 (19)	C11—C6—C7	118.8 (2)
C5—N2—C1	117.3 (2)	C8—C7—C6	119.3 (2)
N2—C1—C2	124.2 (2)	C8—C7—H7	120.4
N2—C1—H1A	117.9	C6—C7—H7	120.4
C2—C1—H1A	117.9	C7—C8—C9	122.7 (2)
C1—C2—C3	117.8 (2)	C7—C8—Cl1	118.76 (18)
C1—C2—H2	121.1	C9—C8—Cl1	118.48 (17)
C3—C2—H2	121.1	C8—C9—C10	117.6 (2)
C4—C3—C2	119.7 (2)	C8—C9—H9	121.2
C4—C3—H3	120.1	C10—C9—H9	121.2
C2—C3—H3	120.1	C11—C10—C9	121.0 (2)
C3—C4—C5	118.2 (2)	C11—C10—H10	119.5
C3—C4—H4	120.9	C9—C10—H10	119.5
C5—C4—H4	120.9	C10—C11—C6	120.6 (2)
N2—C5—N1	114.4 (2)	C10—C11—H11	119.7
N2—C5—C4	122.7 (2)	C6—C11—H11	119.7
N1—C5—C4	122.8 (2)		
			
C5—N2—C1—C2	0.2 (4)	C5—N1—C6—C7	37.1 (4)
N2—C1—C2—C3	0.6 (4)	N1—C6—C7—C8	177.1 (2)
C1—C2—C3—C4	−0.4 (4)	C11—C6—C7—C8	0.8 (3)
C2—C3—C4—C5	−0.5 (4)	C6—C7—C8—C9	−1.4 (4)
C1—N2—C5—N1	−178.1 (2)	C6—C7—C8—Cl1	−178.31 (17)
C1—N2—C5—C4	−1.1 (4)	C7—C8—C9—C10	1.1 (4)
C6—N1—C5—N2	−170.1 (2)	Cl1—C8—C9—C10	178.04 (18)
C6—N1—C5—C4	12.9 (4)	C8—C9—C10—C11	−0.2 (4)
C3—C4—C5—N2	1.3 (4)	C9—C10—C11—C6	−0.3 (4)
C3—C4—C5—N1	178.1 (2)	N1—C6—C11—C10	−176.5 (2)
C5—N1—C6—C11	−146.5 (2)	C7—C6—C11—C10	0.1 (3)

### Hydrogen-bond geometry (Å, °)

**Table:**

*D*—H···*A*	*D*—H	H···*A*	*D*···*A*	*D*—H···*A*
N1—H1···N2^i^	0.88 (1)	2.18 (1)	3.042 (3)	167 (3)

Symmetry codes: (i) −*x*+1, −*y*+1, −*z*+1.

## References

[bb1] Barbour, L. J. (2001). *J. Supramol. Chem.* **1**, 189–191.

[bb2] Bruker (2008). *APEX2* and *SAINT* Bruker AXS Inc., Madison, Wisconsin, USA.

[bb3] Fairuz, M. Z. A., Aiyub, Z., Abdullah, Z. & Ng, S. W. (2008). *Acta Cryst.* E**64**, o1800. 10.1107/S1600536808026317 PMC2960521 21201779

[bb4] Sheldrick, G. M. (1996). *SADABS* University of Göttingen, Germany.

[bb5] Sheldrick, G. M. (2008). *Acta Cryst.* A**64**, 112–122. 10.1107/S0108767307043930 18156677

[bb6] Westrip, S. P. (2009). *publCIF* In preparation.

